# Factors affecting the exposure, vulnerability and emergency medical service capacity for victims of road traffic incidents in Kampala Metropolitan Area: a Delphi study

**DOI:** 10.1186/s12873-016-0112-3

**Published:** 2017-01-07

**Authors:** Joseph Kimuli Balikuddembe, Ali Ardalan, Davoud Khorasani Zavareh, Amir Nejati, Stephen Kasiima

**Affiliations:** 1 Department of Disaster Public Health, School of Public Health, Tehran University of Medical Sciences, Tehran, Iran; 2 Tehran University of Medical Sciences – International Campus, Tehran, Iran; 3 National Institute of Health Research, Tehran University of Medical Sciences, Tehran, Iran; 4 Harvard Humanitarian Initiative, Harvard University, Cambridge, USA; 5 Safety Promotion and Injury Prevention Research Center, Shahid Beheshti University of Medical Sciences, Tehran, Iran; 6 Department of Health in Disaster and Emergency, School of Health, Safety and Engineering, Shahid Beheshti University of Medical Sciences, Tehran, Iran; 7 Department of Clinical Science and Education, Karolinska Institute, Stockholm, Sweden; 8 Department of Emergency Medicine, Imam Khomeini Hospital, Tehran University of Medical Sciences, Tehran, Iran; 9 Directorate of Road Traffic and Road Safety, Uganda Police Force, Kampala, Uganda

**Keywords:** Road traffic incidents, Exposure, Vulnerability, Emergency medical services, Kampala, Uganda

## Abstract

**Background:**

The Kampala Metropolitan Area (KMA) is the fastest developing region in Uganda. Over recent years, this has placed exponential demand on the road sector, which consequently has contributed to rapid growth in motorized vehicles which, predisposes the region to a high risk of road traffic incidents (RTIs). A number of concerted road safety and post-crash management measures to respond to RTIs in the KMA in particular and Uganda as a whole have been undertaken. However, there is a need to greatly improve the measures by better identifying the factors influencing the exposure, vulnerability and emergency medical service (EMS) capacity for RTI victims. The present study seeks to investigate and reveal these factors.

**Methods:**

A Delphi technique employing a questionnaire and involving a multidisciplinary panel of experts was used in three rounds.

**Results:**

The ten (10) most important factors affecting the exposure, vulnerability and EMS capacity for victims of RTIs in the KMA were identified. Socio-cultural, infrastructure and road safety aspects were the factors most identified as affecting the exposure and vulnerability. The absence of a national EMS policy and post-crash care system, as well as the fact that many victims lack health insurance, were noted to be the factors adversely affecting the EMS capacity.

**Conclusions:**

There exists is a real need to substantially reduce the burden of RTIs in KMA, with ultimate goal of saving lives that are being lost needlessly and reducing the impact of injuries and trauma and the economic losses associated with it. This study offers insights into the causes of RTIs and the most appropriate ways of responding to them especially with the establishment and empowerment of predefined and structured EMS systems.

## Background

In recent years, the Kampala Metropolitan Area (KMA) in particular and Uganda as a whole have been registering exponential progress in the road sector. Some roads have been fully or substantially completed; some are under construction and some are due for construction as soon as it becomes possible [1]*.* This crucial progress in the road sector has helped to spearhead the modest economic growth and development in Uganda. However, at the same time it has resulted, in an upsurge of road traffic incidents (RTIs) which contribute to mortality, injury, morbidity, trauma and disability [2–4]*.* RTIs also cost the governments, especially in the low and middle income countries (LMICs) such as Uganda, approximately 3% of their annual Gross Domestic Product (GDP) [5].

The KMA is the fastest developing region in Uganda. It has close proximity to Kampala, the capital city of Uganda, which is the political epicenter and economic hub accounting for 80% of all of Uganda’s industrial and commercial activities [6]. Most of the developed roads in good condition and Emergency Medical Service (EMS) facilities are concentrated in the KMA. Roads from the KMA interconnect with other regions in Uganda (East, West and North) and facilitate international traffic that flows to and from the following neighboring countries: − Burundi, the Democratic Republic of Congo, Kenya, Rwanda, South Sudan and Tanzania. This has resulted in rapid motorization happening in the KMA which has in turn increasing the risk of RTIs.

According to the police records, the highest number of all the registered RTIs in Uganda happens in the KMA. For example, a total of 22,699, 22,461, 22,272, 19,870 and 18,368 RTIs happened in Uganda in 2009, 2010, 2011, 2012 and 2013 respectively. However, 12,099 (53%); 12,152 (54%); 12,136 (54%); ~9935 (50%) and 9651 (53%) respectively were registered in KMA [7–10]. To respond to this, some concerted road safety measures enumerated later in this paper were instigated in the KMA in particular and Uganda in general. Although this is commendable the trend at which RTIs occur in Uganda and the KMA indicates that much more still has to be done. Identifying the exposure and vulnerability factors for victims of RTIs, and the factors affecting the EMS capacity to respond to RTIs needs to be done prior to the implementation of any other measures. Otherwise efforts to improve road safety will be much less effective. This preliminary work is vital if the road safety measures responding to RTIs in the KMA are to have maximum impact. The present study was designed to identifying the factors affecting the exposure (E), vulnerability (V) and emergency medical service capacity (C) for the victims of RTIs in the KMA.

## Method

This study used a Delphi technique employing a questionnaire and involving a multi-disciplinary panel of experts in three rounds. The Delphi method uses a panel of experts to investigate a complex or imprecise issue using a series of structured statements [11, 12]*.* At times, the views of an individual expert can be susceptible to biases so this is why the present study opted for a Delphi approach. Twenty two (22) distinguished experts were purposively contacted to participate in this Delphi study. This was after exploring their profiles and institutions of affiliation which are involved in road safety, EMS and injury prevention. It should however be noted that, no specific number of participants is recommended for Delphi surveys. Only 12 experts participated all the way up to the last Delphi round out of the 16 experts who initially participated in the first round. Their details are indicated in Table 1.

**Table 1:** Details of the experts who completed the last Delphi round

	Details	No participants
Gender		
	Male	9
	Female	3
Professional/employment		
	Road safety policy and traffic accident prevention	7
	Emergency Medical Services	3
	Researcher, academia and consultancy	6
	Regulation of public transport	6
Level of education		
	Bachelor’s Degree	2
	Master’s Degree	10
Years of work		
	<3 years	1
	3–5 years	2
	6–10 years	5
	15–20 years	2
	>20 years	2
Other specialized training		
	Road safety and traffic related course	12
	Accident and disaster emergency care	3
	Others	2

NB: Some experts were involved in more than one profession in terms of employment and training

The selection criteria for the experts was as follows: (a) being recognized as an expert and being knowledgeable on the Delphi topic; (b) having accumulated a wealth of professional experience; (c) holding a key position that requires them to advise and/or oversee issues related to the Delphi topic; (d) being a researcher with recognized research and (e) being affiliated to a reputable institution. The experts selected were government bureaucrats; policy makers; traffic police commanders; academicians and researchers; consultants and emergency care specialists from public and private institutions working on road safety, injury prevention and EMS. A clear explanation about the study was made to them and if they consented (verbally), they were then requested to participate in not less than two Delphi rounds. In each round, the experts were provided with a questionnaire structured in three (3) parts, which solicited their feedback on E, V and C factors as per the study topic. Before distributing the questionnaires for each of the three rounds, all the reviewers carefully reviewed them and where necessary revised them.

### Study area

The KMA covers an area with an approximate 20 km^2^ radius (970 sq. km^2^) in the central part of Uganda. It stretches from Kampala, the capital city of Uganda, to other areas of Mukono, Entebbe, Nansana, Kira and Wakiso [13, 14]. Similar to Kampala, the KMA operates as part of the major business and industrial hub of Uganda - contributing to over 70% industrial production and over 60% of the country’s GDP. Currently, the population in the KMA is estimated at 3.5 million and 60% of all the registered vehicles in Uganda are to be found there [6, 13–15].

### The Delphi procedures

**Round 1:** Initially, 16 experts consented to receiving the open-ended questionnaire which requested their general opinions on the factors influencing the E, V and C for victims of RTIs in the KMA. The experts received the questionnaire in a hard and soft copy form. Answering the questionnaire covered a period between 2 weeks and one and half months. At the beginning, the questionnaire was pretested and reviewed by all the reviewers and where necessary adjusted before being distributed to the experts both in a soft and hard copy forms.

**Round 2:** The experts’ feedback from round one was then reviewed, modified, aggregated and converted into another well-structured questionnaire which consisted of listing a wide range factors affecting the E, V and C for victims of RTIs in the KMA (Table 2). The experts were asked to rate and/or make any possible disagreements on the E, V and C factors presented based on a five Likert type scale (completely agree, agree, no idea, disagree and completely disagree). These questionnaires covered a period between two weeks and one and half months. Where possible, they were also required to state the rationale of their rating and/or offer any additional opinions. The ultimate goal was to derive the ten most important factors according to experts’ ranking. Only 12 experts managed to complete this round. The questionnaires were reviewed by all the reviewers and where necessary adjusted before distributing them to the experts both in a soft and hard copy forms.

**Table 2:** Factors noted in Delphi round one to affect the exposure, vulnerability and EMS capacity for victims of RTI in KMA

	Exposure	Vulnerability	Emergency medical service capacity
1	Lack of driving licenses among drivers	Ignorance and low awareness level on road safety	Crash and injury severity
2	Inadequate driving training regime	Socio demographic factors	Crash type
3	Indiscipline among road users	Driving/or riding incompetency	Number of affected victims
4	Inadequate awareness of road safety laws	Carelessness of pedestrians and cyclists	Time/season in order to determine the deployment
5	Careless road users	Lack of child accompaniment while on roads	Level of survivability
6	Excessive speed	Lack of appropriate driving training	Financial constraints and limited investment in EMS
7	Drinking and driving	Blindness without any guidance	Lack of enough and well - equipped ambulances
8	Ineffective enforcement of traffic laws	Inappropriate infrastructure for pedestrians and non-motorized road users	Access to referral medical facilities and services
9	Low risk perception among road users	Limited interest in road safety sensitization by majority road users	Lack of basic rescue and evacuation skills among lay people
10	Unregulated rise of Boda-bodas	Low risk perception among road users	Occurrence of crash in certain locations
11	Weather conditions	Alcohol and drug influence	Absence of national EMS policy and post-crash care system
12	Poor road engineering design and planning	Poverty leading to unaffordability of safe transport means	Lack of national ambulance network
13	Inefficient public transport system	Use of handheld phones by drivers and other road users while on road	Inadequate pre-crash and post-crash data to inform EMS policies
14	Driving mechanically dangerous vehicles	Riding/ being transported on Boda-bodas	Limited human capacity trained to handle victims
15	Ignoring to use protective safety and visibility gears	Absence of traffic segregation facilities for non-motorized road users	No specialized EMS training courses in medical schools
16	Weak road safety policy in KCCA Act	Weak enforcement of existing traffic laws and regulations	Limited training and knowledge in EMS
17	Poor and inadequate road furniture	Laxity in using protective gears on roads	Lack of emergency call centers for coordination of EMS activities
18	Inadequate pedestrian and cyclist infrastructure	Mixed traffic streams	Unpreparedness among the first responders
19	Lack of segregated lanes AND high traffic mix	Road designs and maintenance not considering vulnerable road users	Lack of health insurance by most of the victims
20	Overpopulation in Kampala	Lack of formal and informal road safety education among road users	Lack of specialized crash and trauma care sections
21	Increased traffic volume and flow	Inadequate regulation of public passenger transport services	In services rotation of EMS staff due to high turn-up of patients
22	Affordability and flexibility of 2 wheeler riders	Inadequate public transport system	Inadequate advocacy for establishing formalized EMS systems
23	Poor traffic lighting	Poor street lighting	Traffic jams preventing timely emergency response to victims
24	Political patronage in road safety enforcement		

**Round 3:** A last version questionnaire, after being validated by all the reviewers, was presented to the experts through email. It consisted of a list in a descending order of the ten highest rated factors from the previous round that were found to be influencing the E, V and C for victims of RTIs in KMA. The overall aim of this round was to elicit the experts’ final judgment, consensus, opinion and or/ related comments specifically relating to the ten highest rated factors. The number of experts who participated in this round remained at 12, and the questionnaire had to be completed within a period of between 2 weeks and one and half months.

Overall, the ten highest rated factors were compared to other factors relayed in an enormity of literature like articles, reports and other publishable information on similar and/or related topics. This helped to further verify and substantiate their impact.

The ethical research requirements and approval were obtained from both the Tehran University of Medical Sciences and Office of the Vice Chancellor for Research and the Uganda National Council for Science and Technology [16, 17].

## Results

**Round one:** In this survey, varying responses and opinions (Table 2) concerning the factors affecting E, V and C for victims of RTIs in the KMA were given by 16 experts who initially participated in the Delphi round one between the months of August and September, 2015.

**Round two:** From all the factors that were given in the first round to have affected E, V and C for victims of RTIs in the KMA, the ten most rated factors (Table 3) were chosen between October and November 2015.

**Table 3:** Ten most rated factors affecting exposure, vulnerability, and EMS capacity for RTI victims in KMA

	Exposure	Vulnerability	Emergency medical service capacity
1	Indiscipline among different road users	Alcohol and substance abuse	Limited staff and well - equipped ambulances
2	Inadequate driving training regime	Lack of appropriate infrastructure for pedestrians and non-motorized road users	Lack of a National EMS policy and post-crash care system
3	Drinking and driving	Absence of traffic segregation facilities for non-motorized road users	Occurrence of crash in certain locations
4	Lack of segregated lanes and high traffic mix	Unaccompanied children on road	Lack of national ambulance network
5	Inadequate pedestrian and cyclist infrastructure	Mixed traffic streams	Lack of public awareness about emergency call centers
6	Ignoring to use protective safety and visibility gears	Demographic aspects (age, sex, peer influence, economic status, and domicile)	Lack of health insurance by most victims to pay for EMS
7	Poor road engineering design and planning	Laxity in using protective gears like reflectors, helmets and seat belts	Lack of specialized crash and trauma care sections
8	Unregulated rise of Boda-bodas	Road designs and maintenance not considering vulnerable road users	Poor and uncoordinated pre and post-crash care
9	Excessive speed	Inadequate regulation of public passenger transport services	Limited trained health care specialist
10	Lack of driving permits/or licenses among drivers	Lack of appropriate driving training	Unpreparedness among first responders

**Round three:** This was the final round which took place between November and December 2015. The experts’ level of agreement and consensus on the order of the ten highest rated factors influencing E, V and C for victims of RTIs in KMA slightly varied from that of round 2. Five experts concurred with the list, but they suggested renaming some of the factors. Two other experts agreed with the list but suggested that to a certain degree the list order should change. They did not say how this should be done. Another 2 experts agreed with the list, but they noted that the exposure factor related to “the unregulated rise in the number of Boda-bodas” (commercial motorcycles) listed needed to be rated much higher. They justified this on the fact that the Boda-bodas are increasingly being registered as the major cause of RTIs in the KMA. The 3 remaining experts never made any comment about the list.

## Discussion

### Main finding

In this Delphi study, the factors that were identified as affecting the exposure and vulnerability of victims to RTIs in KMA were similar and multifactorial in nature. That’s because they were influenced by a number of different socio-cultural, infrastructure and road safety aspects. Those factors most affecting the EMS capacity were the absence of a national EMS policy and post-crash care system as well as the fact that many victims lack health insurance. Given that multidisciplinary experts with varied knowledge; work experience; recognized researches and extramural training were convened together for this study, the factors they identified offer another opportunity to further evaluate and revise the effectiveness of existing road safety measures. The findings also augment a prior advocacy that agitated for establishing a well-structured EMS system to cater for the ever increasing emergency demands being placed on health and medical units beyond even the KMA [2, 4, 18, 19].

### Influence of sociocultural factors to RTIs

In regard to the findings in this Delphi study; the factors related to driver indiscipline; driving under the influence of alcohol and drugs; failure to use adequate safety equipment (seat belts, helmets, visibility reflectors and child restraints) and excessive speeding were often induced by different road users’ cultures, behaviors and perceptions. Other factors were demographically attributed to age, peer influence and the economic status. Some of these factors were identified in other studies to contribute to the underlying causes of RTIs in LMICs including a systematic review study on the trend of RTIs in Kampala and Uganda at large [20–24]. Similar findings in other studies also showed how introverted and extroverted cultural attitudes, behaviors and perceptions were stronger predictors of involvement in RTIs both in high income countries and LMICs, which included Uganda [25, 26]. However, the contribution of these factors to RTIs were exacerbated by the other factors like the rapid motorization and unprecedented population growth in the KMA.

### Road infrastructure

The aforementioned road sector in Uganda has been progressing especially in the KMA with the benefits of the region having the most roads in fairly good condition. Approximately 1300 km^2^ (43%) of paved roads exist in the KMA out of 3000 km^2^ of total paved roads in Uganda [6, 13, 14]. Despite this advantage the highest number of RTIs in Uganda were recorded in the KMA as mentioned above. Aside other challenges, the conditions of many roads in the KMA are still poor and others lack vital traffic safety and pedestrian infrastructure. For instance, Kampala city has approximately 1200 km^2^ of road network but only 20% of it is in fair condition [6].

Again, the majority of roads in the KMA lack necessary traffic signs and pedestrian facilities such as speed humps, warning signs, demarcated bus-stops, zebra crossing, footbridges and crosswalks as well as pedestrian reflectors. Yet these traffic signs and facilities are required for regulating the traffic flow and alerting road users about potential hazards along roads which ultimately help to reduce RTIs [27]*.* This validates some of the E and V factors that were underscored to affect victims of RTIs in the KMA by the experts because their causes mostly emanate from road design and construction challenges. They include: lack of segregated lanes; inadequate road furniture; poorly planned-engineering; mixed traffic streams; poorly designed roads and the absence of traffic segregation facilities for non-motorized road users. These are similar with other findings in most of the LIMCs [20, 28, 29], which are attributed to a lack of a systematic approach in preventing RTIs [30, 31].

### Road safety

It is pleasing to report that over the recent years, multisectoral road safety measures were instigated to respond to the heavy burden of RTIs. These included the deployment of more traffic and highway police along roads to police for road violations; efforts to increase public awareness and sensitization; greater coordination efforts and proactive road safety audits [7–10]. They have also been augmented by the enacting of regulations covering driving permits (2005); weighing bridges (2009) and driving schools and driver instructors (2010). The Ministry of Works and Transport in Uganda has been taking a lead role in their implementation in collaboration with other stakeholders [32, 33]*.*

The above road safety measures are in-tandem with the national, regional and international road safety frameworks based on the Uganda National Road Safety Action Plan and United Nations General Assembly Action for the Road Safety Action 2010–2020 and are to be commended [33, 34]*.* However, much more is still needed to achieve effective implementation and enforcement of road safety in the KMA considering some factors which were noted in the Delphi surveys. These include: an inadequate driver training regime; ignoring use of protective equipment; drinking and driving; excessive speed; lack of driving permits; and the inadequate regulation of public passenger transport services. Most of these factors have been also highlighted by the World Health Organization, and they are also in line with the findings in another study [29, 35].

### Distribution of victims

Based on RTI records all the road users (pedestrians, passengers, riders of motorized 2 or 3 wheelers, motor and pedal cyclists and drivers of different vehicles) in the KMA are susceptible to being involved in RTIs [7–10]. Some road users are vulnerable to RTIs largely because of their own reckless actions or inactions. Pedestrians and passengers, however, are commonly much more vulnerable to becoming innocent victims of RTIs compared to other road users [7–10, 36–38]. The riders and passengers of the 2 wheeler Boda-bodas (commercial motorcycles) have also increasingly become vulnerable to RTIs.

Of recent, the numbers of Boda-bodas have been exponentially rising to the point that it now predominates the public transport system of the KMA and Uganda [3, 24, 36, 38, 39]. They are mainly manned by the youths who seek to make-ends-meet. Boda-bodas have been dubbed a ‘silent killer’ and they not only cause serious injuries and fatalities but also a significant economic burden [3, 19, 36–40]*.* Some of the reasons attributed to this include failure to wear and use protective safety and visibility gear and equipment (helmet and reflectors); failure of riders to comply with the traffic signals; riding at exorbitant speeds where it is unnecessary and dangerous. Researcher further indicates that the severity of injuries of the victims involved in Boda-boda crashes in Kampala city is greatly increased particularly by the failure of both motorcyclists and their passengers to wear protective gear [3, 37–39]*.* It is therefore necessary to rank the unregulated rise of the Boda-bodas higher among the factors exposing victims to RTIs in KMA as some experts suggested.

### Emergency medical services

The absence of a national EMS policy and post-crash care system are compounded by other factors known to affect the EMS capacity. This problem has been identified before in various studies. One of the studies was concerned about the lack of a well-functioning pre-hospital emergency and trauma care system which can be widely accessible [19]*.* Two studies which assessed the pre-hospital care for victims of RTIs observed that it’s often left to police with the help of community leaders, taxi drivers and lay people who are ill-equipped and untrained to deliver suitable emergency medical care [4, 18]*.* Other studies noted that due to the absence of a pre-hospital emergency care system in Kampala, many patients arrive too late and those with serious injuries may die before they receive the emergency care they need. Others die immediately upon arrival to accident and emergency units due to poor emergency care they received before [2, 41]*.*

Ideally, a definitive and well-planned emergency care system needs to be urgently put in place. That should greatly help to significantly reduce deaths, injuries, morbidity and disability adjusted life years (DALYS) caused by RTIs [42]*.* That would facilitate quick life-saving care and improve treatment which reduces and prevents life threatening injuries due to the potential effects of RTIs. The establishment of a well-planned and coordinated EMS system based on patient needs over time has been previously called for in different forums. As a result of this advocacy the Uganda National Ambulance Services (UNAS) [43], was established to coordinate ambulance-related activities in Uganda. This was a positive step forward, however, much is still to be done considering some of the factors identified through the Delphi process that affect the EMS capacity.

### Health insurance for victims of RTIs

At times, depending on the injury severity, the emergency care for RTI victims can be complex requiring more resources and quick responses compared to those needed by other patients. It can thus, require and precipitate hurried and costly resource deployment [44]*.* However, this remains a challenge in many LMICs and Uganda is no exception, where the structured EMS systems are not yet in place. In a study on unintentional childhood injury in Kampala, it was found that only 3% of victims were treated on insurance [36]. Similarly, another study noted that less than 5% of patients in Kampala were brought to health facilities by the few existing ambulances, most of which were privately run and prohibitively expensive [4]*.* Notwithstanding other factors, the findings of both studies could be attributed to the lack of a mandatory national health insurance policy in Uganda. Currently, fairly good EMSs in Kampala are privately offered, but at a premium. Free EMS is available in the government funded public health facilities. The poor and low-income earners involved in RTIs run the risk of not receiving quality EMS if they can’t easily access it. Therefore, since RTIs are unpredictable events, there’s a great need for Uganda to come-up with a mandatory national health insurance policy because it was identified in this study to be one of the main factors affecting the EMS capacity. A national health insurance system, if adopted, should also consider subsidizing the EMS to cater for all patients’ needs, especially for poor and low income earners.

### Study strength and limitations

A noteworthy strength of this study is that it managed to convene multidisciplinary experts to deliberate on a common public health challenge affecting the KMA in particular and Uganda at large. Fortunately, 50% of the panelists were members of Uganda National Council of Road Safety. Therefore the factors that were listed to affect the E, V and C are dependable and oriented by the experts’ across multiple professions; educational levels; work experiences; research; and extramural training beyond the arena of road safety and EMS.

On the other hand, the experts’ opinions should not be conclusive, and some caution is advisable while interpreting them based on two reasons. First, the definition of an expert is subjective and the criteria, which was used to select the experts, was to some degree influenced by references and networks. This might have accrued some degree of bias. Second, the Delphi method only explores areas of concern raised by the panel of experts, and so other important areas other than the main topic of discussion might have been overlooked.

## Conclusion

This study explored the factors affecting the E, V and C for victims of RTIs in KMA through the Delphi technique which involved a number of multidisciplinary experts on road safety, injury prevention and EMS. The socio-cultural, infrastructure and road safety aspects were identified as factors influencing the victims’ exposure and vulnerability to RTIs. The absence of a national EMS policy and post-crash care system, as well as the fact that many victims lack health insurance, were noted to be the factors adversely affecting the EMS capacity. Although a multiplicity of road safety measures are in place to reduce the burden of RTIs, there exists a real need to substantially reduce the burden of RTIs in the KMA, with ultimate goal of preventing needless deaths; reducing the impact of injuries and trauma and the economic losses associated with it. An urgent need is similarly required to establish and empower of predefined and structured EMS systems. This study offers some useful insights on the factors that were identified to affect the E, V and C for victims of RTIs in the KMA drawn from a panel of experts, and should guide any measures taken to respond to the burden of RTIs in the KMA and elsewhere in Uganda.

## Abbreviations

C: Emergency medical capacities
E: Exposure
EMS: Emergency medical services
KMA: Kampala Metropolitan Area
LMICs: Low and middle-income countries
RTIs: Road traffic incidents
TUMS: Tehran University of Medical Sciences
UNCST: Uganda National Council of Science and Technology
V: Vulnerability
