# Erratum to: Factors affecting the exposure, vulnerability and emergency medical service capacity for victims of road traffic incidents in Kampala Metropolitan Area: a Delphi study

**DOI:** 10.1186/s12873-017-0115-8

**Published:** 2017-01-25

**Authors:** Joseph Kimuli Balikuddembe, Ali Ardalan, Davoud Khorasani-Zavareh, Amir Nejati, Stephen Kasiima

**Affiliations:** 1 Department of Disaster Public Health, School of Public Health, Tehran University of Medical Sciences, Tehran, Iran; 2 Tehran University of Medical Sciences – International Campus, Tehran, Iran; 3 National Institute of Health Research, Tehran University of Medical Sciences, Tehran, Iran; 4 Harvard Humanitarian Initiative, Harvard University, Cambridge, USA; 5 Safety Promotion and Injury Prevention Research Center, Shahid Beheshti University of Medical Sciences, Tehran, Iran; 6 Department of Health in Disaster and Emergency, School of Health, Safety and Engineering, Shahid Beheshti University of Medical Sciences, Tehran, Iran; 7 Department of Clinical Science and Education, Karolinska Institute, Stockholm, Sweden; 8 Department of Emergency Medicine, Imam Khomeini Hospital, Tehran University of Medical Sciences, Tehran, Iran; 9 Directorate of Road Traffic and Road Safety, Uganda Police Force, Kampala, Uganda

## Erratum

The original article [1] contained an error whereby the author Davoud Khorasani-Zavareh's name was not presented correctly.

This error has now been corrected.
